# Biodiversity of aquatic organisms in the Mekong Delta, Vietnam

**DOI:** 10.3897/BDJ.11.e105314

**Published:** 2023-10-24

**Authors:** Dmitriy G Seleznev, Cu Nguyen Dinh, Truong Ba Hai, Evgeniia P. Karpova, Duong Thi Kim Chi, Dmitriy B. Kosolapov, Natalya G. Kosolapova, Mikhail I. Malin, Inga P. Malina, Le Quang Man, Alexander A. Prokin, Irina Yu. Prusova, Andrey N. Sharov, Svetlana V. Statkevich, Alexander I. Tsvetkov, Yuriy G. Udodenko, Viktor V. Zakonnov, Svetlana M. Zhdanova, Alexander V Krylov, Alexei V. Tiunov

**Affiliations:** 1 Papanin Institute for Biology of Inland Waters, Russian Academy of Sciences, Borok, Yaroslavl region, Russia Papanin Institute for Biology of Inland Waters, Russian Academy of Sciences Borok, Yaroslavl region Russia; 2 Southern Branch of the Joint Russia-Vietnam Tropical Science and Technology Research Center, Ho Chi Minh City, Vietnam Southern Branch of the Joint Russia-Vietnam Tropical Science and Technology Research Center Ho Chi Minh City Vietnam; 3 A.O. Kovalevsky Institute of Biology of the Southern Seas, Russian Academy of Sciences, Moscow, Russia A.O. Kovalevsky Institute of Biology of the Southern Seas, Russian Academy of Sciences Moscow Russia; 4 A.N. Severtsov Institute of Ecology and Evolution, Russian Academy of Sciences, Moscow, Russia A.N. Severtsov Institute of Ecology and Evolution, Russian Academy of Sciences Moscow Russia

**Keywords:** database, Mekong Delta, southern Vietnam, aquatic organisms, macrozoobenthos, phytoplankton, zooplankton, microorganisms, fish, hydrology, bottom sediments

## Abstract

**Background:**

The Mekong River is the 10^th^ largest river in the world. It is recognised as the most productive river in Southeast Asia and economically essential to the region, with an estimated 60-65 million people living in the lower Mekong Basin. The Mekong Delta within Vietnam is considered a highly vulnerable ecosystem under threat from increasing anthropogenic pressure, such as dam construction and, as a consequence, the Delta is sinking and altering the natural hydrological cycle. Dams also lead to eutrophication and pollution of downstream water from regulated water flux and water stagnation. Another threat is climate change coupled with the lower rainfall, which could lead to an increased risk of drought in the Mekong Delta Basin. Thus, these project data represent an important baseline reference. The ecological health of the Mekong Delta’s environment, as indicated by the quality and availability of its water and biological resources, largely determines the economic and social development of the region, which produces about half of the agriculture and aquaculture products of Vietnam.

**New information:**

This paper reports quantitative data on the biodiversity of six groups of aquatic organisms: bottom and pelagic fish, macrozoobenthos, microorganisms, phyto- and zooplankton in the Mekong Delta within Vietnam, as well as data on the physicochemical parameters of water and bottom sediments. The data were collected during 2018-2022 as part of the Ecolan E-3.4 programme within the framework of the research plan of the Joint Russian-Vietnamese Tropical Research and Technological Center. All presented datasets are published for the first time.

## Introduction

The Mekong River is the main waterway of the Indochina Peninsula and is heavily relied on as the core resource for sustainable socio-economic development for most of the countries located in its densely populated Basin. While the Mekong Delta makes up only about 12% of the territory of Vietnam, it annually accounts for about 55% of its rice production, 70% of aquaculture production and more than 50% of fishery production (Mekong River Commission 2020, General statistics office 2021). The rapidly growing population of Southeast Asia, coupled with the developing economy of the region, requires the involvement of an increasing amount of water, energy and biological resources. In the lower reaches of the Mekong River, the increased anthropogenic load in the river basin has been acutely felt in recent decades, against the backdrop of pronounced climatic changes (Tuan and Chinvanno 2011, Thilakarathne and Sridhar 2017). In addition to the recent changes in the water regime of the Delta, the sanitary and ecological state of the river is at risk from surface water run-off of pesticides from agricultural lands, sewage discharge, domestic waste and the transboundary transport of pollutants from upriver (the Mekong watershed includes land in China, Laos, Thailand and Cambodia) (Li et al. 2013). Furthermore, large-scale hydro-technical development, including the construction of protective dams and agricultural irrigation canals, has a negative impact on the lower reaches of the Mekong River, especially in the Delta of Vietnam (Winemiller 2016, Le_Meur et al. 2020). These factors, along with the increased intensity of fisheries and aquaculture, pose a serious threat to the biological diversity of the region, in which the lower Mekong is second only to the Amazon and Congo Rivers (Campbell 2012). The inventory of biological diversity and biological resources of the region is far from complete; every year, dozens of species new to science are described. The presented data were collected in 2018-2022 as part of a comprehensive study aimed at assessing the current state of biological resources and the trophic base of commercial fish and invertebrates.

## General description

### Purpose

This is a comprehensive study of the biodiversity of the Mekong River's lower reaches under changing environmental conditions. We present data concerning:

- Hydrology and hydrochemistry of the Mekong River.
- Composition of plankton communities.
- Composition of pelagic and demersal fish and macrozoobenthos.
- Spatial structure of the fish populations in the Mekong Delta.

### Additional information

The work was carried out according to the research plan of the Joint Russian-Vietnamese Tropical Research and Technological Center (Tropical Center). The Tropical Center is a large multidisciplinary institution that conducts long-term research aimed at studying the structure and functioning of natural and transformed tropical ecosystems.

## Project description

### Title

Ecosystem of the Mekong River in the context of global climate change and anthropogenic impact

### Personnel

Alexander I. Tsvetkov - stations hydrological data, Evgeniya P. Karpova and Svetlana N. Statkevich - bottom trawls hydrological data, bottom fish data, Yuriy G. Udodenko and Viktor V. Zakonnov - bottom sediments data, Alexander A. Prokin and Alexander I. Tsvetkov - macrozoobenthos data, Andrey N. Sharov - phytoplankton data, Dmitriy B. Kosolapov and Natalya G. Kosolapova - microorganisms data, Mikhail I. Malin and Inga P. Malina - pelagic trawls parameters and pelagic fish data, Svetlana M. Zhdanova and Irina Yu. Prusova - zooplankton data, Cu Nguyen Dinh, Truong Ba Hai, Le Quang Man, Duong Thi Kim Chi - investigation and resources, Dmitriy G Seleznev - data curation and text writing, Alexander V. Krylov and Alexei V. Tiunov - supervision.

### Study area description

**Figure 1.**
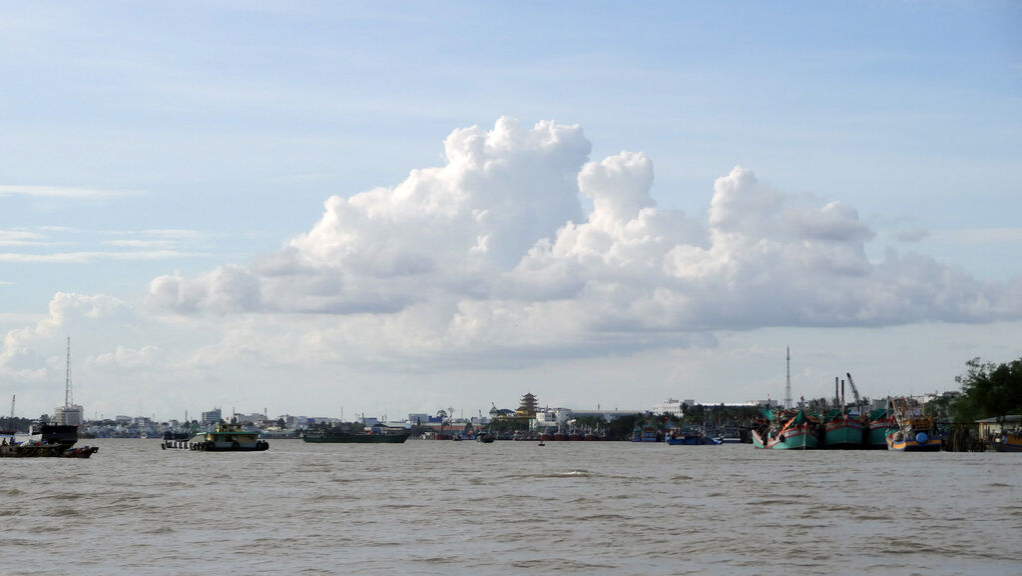
Mekong Delta near My Tho city. Photo by Alexander A. Prokin.

**Figure 2.**
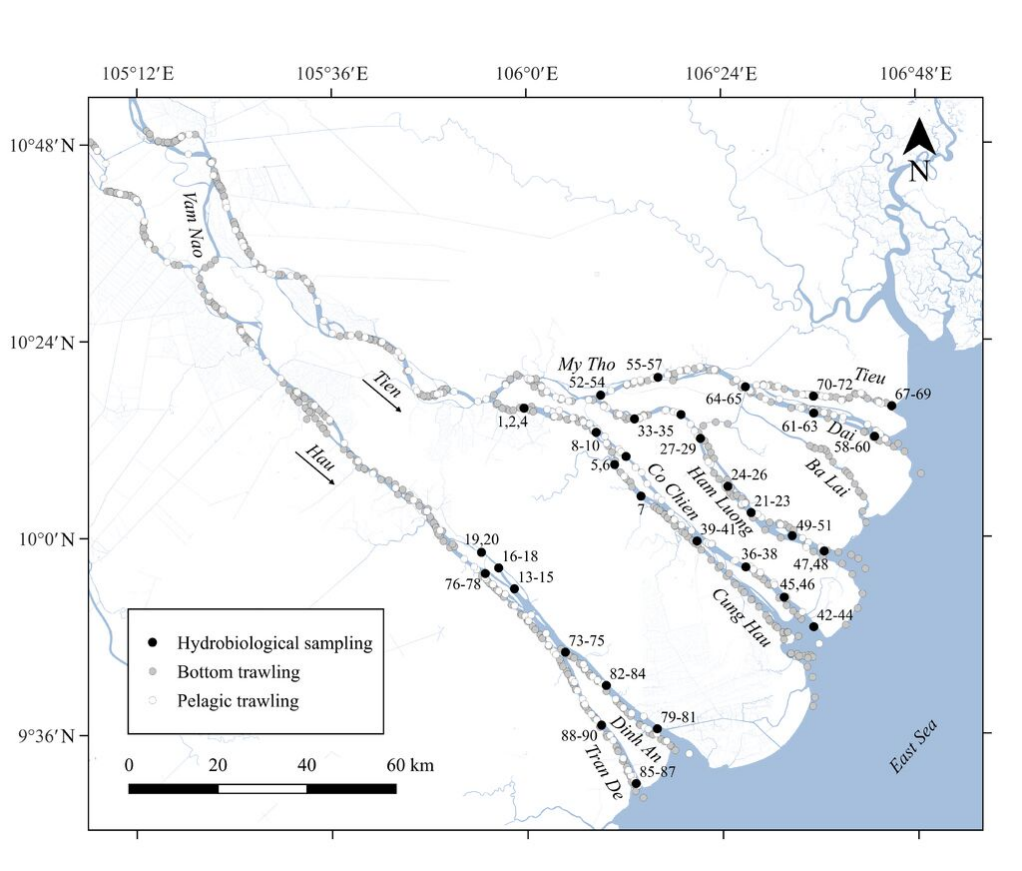
Mekong Delta map. Created by Mikhail I. Malin

The studies were carried out in from 2018 to 2022 in the Mekong Delta, Vietnam (Figs 1, 2).

### Funding

The main funding was obtained via the Joint Vietnamese-Russian Tropical Research and Technological Center (Ecolan E-3.4 topic). Additional funding was obtained from the Ministry of Science and Higher Education of the Russian Federation (projects no. 121051100109-1, 121030100028-0, 121051100102-2, 121051100099-5 and 121051100104-6).

## Sampling methods

### Sampling description

A total of 926 water samples were taken to measure hydrological water parameters, while 70 bottom sediments, 36 macrozoobenthos, 35 phytoplankton, 50 microorganism and 73 zooplankton samples were taken from 89 static sampling points. For trawling samples, a total of 535 samples of fish and water (for additional hydrological parameter measurements) were taken by bottom trawling, while 232 samples of fish were taken by pelagic trawling (see summary in Table 1).

**Water hydrological parameters measurements**.

Water parameters (temperature, oxygen content and saturation, electrical conductivity, total salt content, salinity) were measured using a YSI ProPlus multi-parameter sonde (YSI Inc. USA) over the entire water column with a resolution of 1 m. Turbidity at surface horizons was determined using a Hach 2000P Portable Turbidimeter (Hach Inc. USA) according to standard methods. pH and redox potential were determined in the surface water layer using a Hanna HI 98121 hand probe (Hanna Instruments, USA). The flow velocity of the surface water was measured using a microcomputer speedometer-flowmeter MKRS (GVMNPP VODKOSMOS, Belarus), as well as using the Global Water Flow Probe - FP211 (YSI Inc. USA).

**Bottom trawls hydrological parameter measurements**.

Various physicochemical measurements were carried out at all sampling sites. Water samples for analysis were taken using a bathometer. The measurements were carried out at the end of trawling. A TDS-3 multimeter (HM Digital, Inc.) and a PAL-06S refractometer (ATAGO Co, Ltd.) were used for measuring water salinity. Subsequently, using a HANNA HI 9146-04 oximeter, a HI-9813-6 pH/EC/TDS/°C multimeter and a YSI ProPlus (YSI Inc. USA) multi-parameter probe, the content of dissolved oxygen, pH, electroconductivity and total dissolved solids were measured in the surface and near-bottom horizons. In February–June 2020, water transparency was additionally measured using a Secchi disk.

**Bottom fish assessment**.

Catching fish was carried out using a standard fishing trawl with a metal frame. The width of the trawl frame was 4 m and the height was 0.4 m. The length of the trawl bag was 12 m and the mesh size was 10 mm. The trawl was towed along the bottom from a motorised fishing boat. During trawling, their duration was noted. The coordinates of the beginning and end of the work were determined using GPS. Based on these data, the average trawling speed was determined. Using a Garmin STRIKER echo sounder, the average depth of each haul was determined.

After the trawl was lifted on to the deck of the boat, the catch was analysed and fish were separated. Animals were placed in coolers with ice and further studies were carried out under laboratory conditions. For fish, taxonomic affiliation was established at the family level (Rainboth 1996, Tran et al. 2013, Tri 2015) and the number and total mass of representatives of each family were determined using calibrated weights.

The mass of macroplastic fragments and plant substrate was determined in the composition of trawl catches. To do this, these components were manually removed from the trawl, washed with water, dried and weighed on a calibrated balance.

**Bottom sediments assessment.**

Samples of surface layer sediments (0–5 cm) were taken in December 2018 and April 2019 using a gravity-type corer. Three subsamples were collected at each site, mixed and placed in plastic bags pre-washed with 10% nitric acid. During sampling, the outermost layer of the sediments was discarded to avoid possible contamination from the sampler. Before analysis, samples were air-dried at room temperature, ground in a porcelain mortar and passed through a 1-mm sieve. Grain size determination was performed using an electromagnetic sieving analyser (Alfred Fritsch & CO, Germany) and wet sieving through sieves of different diameters. Organic matter content was determined as loss of ignition: 2 g samples were placed in an oven and heated to 600°C. The total mercury concentration was determined by pyrolysis using direct thermal decomposition atomic absorption spectrometry (RА-915, Lumex, Russia) using a pyrolytic attachment PYRO. This method of analysis does not require additional chemical preparation of samples.

**Macrozoobenthos assessment**.

Macrozoobenthos was sampled with a Petit Poinar Grab, All 316 (producer: Wildlife Supply Company, capture area 0.023 m^2^; two liftings of sediments). Sediments were filtered through a sieve with a mesh size of 200 × 200 μm and all macroinvertebrates were preserved in 96% alcohol immediately on the boat. The fresh weight of specimens was determined after the removal of surface moisture (drying on a filter paper until wet spots disappeared) in a laboratory using a WT-100 torsion balance (weighing accuracy of 0.1 mg). Invertebrates were determined using several keys and taxonomic revisions (Brandt 1974, Burch 1980, Barnard and Barnard 1983, Glasby 1999, Mekong River Commission 2006). Mollusca were deposited in the collection of the A.N. Severtsov Institute of Ecology and Evolution RAS (Moscow), with other groups in the water invertebrates collection of the Papanin Institute for Biology of Inland Waters of the Russian Academy of Sciences (IBIW, Borok, Yaroslavl Region, Russia).

**Phytoplankton assessment**.

The research was conducted in December (at the beginning of the dry season) of 2018 in three tributaries of the Mekong Delta: Ham Luong, Co Chien and Hau or Bassac. The samples were collected from the upper horizon using Rutner's bathometer. The water samples were preserved with 1% Lugol’s solution and concentrated using direct filtration with low pressure one after another through membrane filters of 5 μm diameter and then filters of 1.2 μm diameter. Phytoplankton cells in each sample were counted and identified to species level using a Nageotte chamber (0.02 ml) under an optical microscope BiOptic B-200 (BiOptic Russia, Moscow) at 400× and 600× magnification. The phytoplankton found was verified in the AlgaeBase (Guiry and Guiry 2022). Species cell size and biovolumes were measured using the common counting-volumetric method (Hillebrand et al. 2002). Species comprising over 10% of the total biomass of plankton algae were identified as the dominant species.

**Microorganisms assessment**.

Water was taken from the surface layer of the river using a Van Dorn sampler and immediately placed into 60 ml sterile plastic vials. Water samples were fixed with 40% formaldehyde to a final concentration of 2%, stored in the dark at 4°C and processed in the laboratory for 2 months.

The abundance and size of bacteria, picoplankton, heterotrophic and phototrophic flagellates were determined by epifluorescence microscopy using an Olympus BX51 microscope (Japan) with an image analysis system at a magnification of 1000 times (Porter and Feig 1980, Caron 1983, Tsuji et al. 1986). The volumes of microbial cells were calculated using the formulae for the volumes of a cylinder, sphere or ellipsoid. The wet microbial biomass was calculated by multiplying their abundance by the average cell volume. The carbon content in bacterial cells (C, fg C/cell) was calculated using the following allometric equation: C = 120 × V^0.72^ (Posch et al. 2001). The coefficients of 200 and 220 fg C/μm^3^ were used to convert picoplankton and flagellate biomass into carbon units (Borsheim and Bratbak 1987, Weisse 1993).

Heterotrophic flagellates were identified to species or genus in non-fixed water samples using phase-contrast microscopy and an image analysis system. The detected flagellates were diagnosed by morphological characters and features of their behaviour (Vørs 1992).

**Zooplankton assessment**.

Zooplankton (Rotifera, Cladocera and Copepoda, larvae of Mollusca) samples were taken by a modified weighted Juday net (inlet diameter 0.18 m, 64 μm mesh size) completely from the bottom to the water surface. In the areas with strong flow, 47.5–100 l of water were filtered through the net. All samples were immediately fixed in 4% formalin. The volume of subsamples was 1–4 ml (from 50 or 100 ml samples). In cases the density of specimens was low, the zooplankton counted in the whole samples. The metazoan zooplankters were identified and counted using a stereomicroscope (Micromed MC-2 Zoom, Russia) with 40x magnification and a Bogorov counting chamber. The zooplankton specimens were identified to the lowest taxonomic level possible using a microscope (Biomed 1, Russia). Species were identified according to Shirota (1966), Dang et al. (1980), Koste and Shiel (1987), Reddy (1994), Shiel (1995), Khoi (2001), Ueda and Reid (2003), Kotov et al. (2009), Sinev (2016). The zooplankton found were verified in the WoRMS database (Horton et al. 2022). The individual wet weights of zooplankton specimens were estimated from average lengths according to Ruttner-Kolisko (1977), Balushkina and Vinberg (1979).

**Pelagic fish assemblage composition assessment with a mid-water trawl.**

Fish samples were collected using a mid-water trawl with a 12 m horizontal opening and 8 mm mesh size in the codend. The trawl was suspended in the water with the floats attached to the trawl doors by bridles. The selection of sampling layers was accomplished by adjusting the bridle length from 1 to 7 m. The trawl was towed for approximately 20 min at each sampling site. Effort features (geographic coordinates of trawling start and finish; vessel speed; trawled distance; minimal, maximal and mean bottom depth values) were documented from recordings of an echosounder “Simrad EK80” (Kongsberg Maritime, Norway) interfaced with a satellite navigation receiver. Catch was weighed, sorted and counted onboard. Identification of fish families was made using common taxonomic keys (Rainboth 1996, Tran et al. 2013). The relative abundance of fish families at sampling sites was estimated using CPUE (catch per unit effort, ind./h) value.

## Geographic coverage

### Description

Static samples from 89 locations were taken on the Tien branch from the City of Vinh Long and on the Hau branch from the City of Tra On, to the confluence of the branches into the East Vietnamese Sea. Bottom and pelagic trawling were conducted in the Mekong Delta from the Cambodian border to the mouth of the river (Fig. 2).

## Temporal coverage

**Data range:** 2018-5-01 – 2022-5-23.

## Usage licence

### Usage licence

Creative Commons Public Domain Waiver (CC-Zero)

## Data resources

### Data package title

Quantitative data of aquatic organisms and water hydrological parameters in the Mekong Delta, Vietnam

### Number of data sets

9

### Data set 1.

#### Data set name

Stations positions and water hydrological parameters

#### Data format

Semi-colon delimited CSV file

#### Description

These data describe the geographical position of the static sampling stations as well as the dated physicochemical parameters of water at different depths. In total, 89 stations were studied. This dataset will be useful for analysing samples taken at these stations. The dataset is in Suppl. material 1.

**Data set 1.** 

Column label	Column description
Station	Station number.
Waterbody	River and branch name.
Latitude	Latitude.
Longitude	Longitude.
Position	Position in watercourse: right ripal, medial or left ripal. The term «ripal» is used for riparian (shore) part of the river.
Dist2mouth	Distance to mouth, km.
Datetime	Date and time of sampling.
Depth	Depth, m.
T	Temperature, C.
DOX	Dissolved oxygen, mg/l.
DOXS	Dissolved oxygen saturation, %.
Cond	Conductivity, μS/cm.
SCond	Specific conductance 25 C, μS/cm.
TDS	Total dissolved solids, mg/l.
Sal	Salinity, ppt.
Dens	Density.
Turb	Turbidity, NTU.
Vel	Current speed, m/sec.
pH	pH.
Trans	Transparency, m.
ORP	Oxidation reduction potential, mV.

### Data set 2.

#### Data set name

Bottom trawls location and water hydrological parameters

#### Data format

Semi-colon delimited CSV file

#### Character set

UTF-8

#### Description

These data describe spatial and temporal characteristics of 535 bottom trawls as well as the hydrological parameters of water at trawling points. This dataset will be useful for analysing the benthic fish data. The dataset is in Suppl. material 2.

**Data set 2.** 

Column label	Column description
Trawl	Trawling name code.
Waterbody	River and branch name.
SLatitude	Trawling start latitude.
SLongitude	Trawling start longitude.
ELatitude	Trawling end latitude.
ELongitude	Trawling end longitude.
Length	Trawling length, m.
Datetime	Trawling date and start time.
Depth	Trawling mean depth, m.
TSpeed	Trawling speed, m/min.
TDuration	Trawlng duration, min.
s.T	Surface temperature, C.
b.T	Bottom temperature, C.
s.S	Surface salinity, per mille.
b.S	Bottom salinity, per mille.
s.DOX	Surface dissolved oxygen, mg/l.
b.DOX	Bottom dissolved oxygen, mg/l.
s.DOXS	Surface dissolved oxygen saturation, %.
b.DOXS	Bottom dissolved oxygen saturation, %.
s.SPS	Surface specific conductance 25 C, µS/cm.
b.SPS	Bottom specific conductance 25 C, µS/cm.
s.TDS	Surface total dissolved solids, mg/l.
b.TDS	Bottom total dissolved solids, mg/l.
s.pH	Surface pH.
b.pH	Bottom pH.
Trans	Transparency, cm.
PlastD	Macroplastic debris, kg.
PlantD	Plant debris, kg.

### Data set 3.

#### Data set name

Bottom fish

#### Data format

Semi-colon delimited CSV file

#### Character set

UTF-8

#### Description

These data describe bottom trawl catches represented by abundance and biomass of 49 fish families belonging to 19 orders of classes Chondrichthyes and Actinopterygii. Taxonomic membership of ~ 88,000 fish individuals from 535 trawls were identified at the family level. The dataset is in Suppl. material 3.

**Data set 3.** 

Column label	Column description
Trawl	Trawl name code.
Waterbody	River and branch name.
Depth	Depth, m.
Datetime	Sampling date and time.
Taxa	Taxa name.
Number	Number, ind.
Biomass	Biomass, g.

### Data set 4.

#### Data set name

Bottom sediments

#### Data format

Semi-colon delimited CSV file

#### Character set

UTF-8

#### Description

These data describe the size and type of bottom sediments as well as total organic matter content and mercury concentration at 70 stations. The dataset is in Suppl. material 4.

**Data set 4.** 

Column label	Column description
Station	Station number.
Period	Sampling period.
Depth	Depth, m.
Sed1	Sediments fraction 1.0 - 0.5 mm, %.
Sed2	Sediments fraction 0.5 - 0.1 mm, %.
Sed3	Sediments fraction 0.1 - 0.05 mm, %.
Sed4	Sediments fraction 0.05 - 0.01 mm, %.
Sed5	Sediments fraction < 0.01 mm, %.
Gravel	Gravel fraction > 1.0 mm, %.
Sand	Sand fraction 10 - 0.05 mm, %.
Clay	Silt and clay fraction < 0.05 mm, %.
mean_D	Mean fraction diameter, mm.
TOM	Total organic matter, %.
Hg	Hg, mg/kg.

### Data set 5.

#### Data set name

Macrozoobenthos

#### Data format

Semi-colon delimited CSV file

#### Character set

UTF-8

#### Description

These data describe abundance and biomass of benthic invertebrates of five phyla: Annelida, Arthropoda, Nemertini, Echinodermata and Mollusca. In total, 56 species were identified at 29 stations. The dataset is in Suppl. material 5.

**Data set 5.** 

Column label	Column description
Station	Station number.
Waterbody	River and branch name.
Depth	Depth, m.
Period	Sampling period.
Phylum	Phylum name.
Taxa	Taxa name.
Number	Number, ind/m^2^.
Biomass	Biomass, g/m^2^.

### Data set 6.

#### Data set name

Phytoplankton

#### Data format

Semi-colon delimited CSV file

#### Character set

UTF-8

#### Description

These data describe the biomass of six groups of phytoplankton: Cyanobacteria, Cryptophyta, Dinophyta, golden, green and diatom algae at 35 stations. The total phytoplankton abundance at the stations is also provided. The dataset is in Suppl. material 6.

**Data set 6.** 

Column label	Column description
Station	Station number.
Waterbody	River and branch name.
Taxa	Taxa name.
Biomass	Biomass, mg/l.
Abundance	Abundance, 10^3^ cells/l.

### Data set 7.

#### Data set name

Microorganisms

#### Data format

Semi-colon delimited CSV file

#### Character set

UTF-8

#### Description

These data describe cell abundance, mean cell volume, biomass and carbon biomass of bacteria, picophytoplankton, phototrophic and heterotrophic nanoflagellates. In total, 50 stations were sampled. The dataset is in Suppl. material 7.

**Data set 7.** 

Column label	Column description
Station	Station numberю.
Waterbody	River and branch name.
Depth	Depth, m.
Taxa	Taxa name.
Abundance	Cells abundance, 10^3^ cell/ml.
Biomass	Biomass, mg/m^3^.
C.Volume	Mean cell volume, μm^3^.
C.Biomass	Carbon biomass, mgС/m^3^.

### Data set 8.

#### Data set name

Zooplankton

#### Data format

Semi-colon delimited CSV file

#### Character set

UTF-8

#### Description

These data describe the biomass and total abundance of five groups of zooplankton: Rotifera, Cladocera, Copepoda and veligers of Gastropoda and Bivalvia at 73 stations. The dataset is in Suppl. material 8.

**Data set 8.** 

Column label	Column description
Station	Station number.
Waterbody	River and branch name.
Period	Sampling period.
Taxa	Taxa name.
Number	Nunber, ind/m^3^.
Biomass	Biomass, mg/m^3^.

### Data set 9.

#### Data set name

Pelagic fish

#### Data format

Semi-colon delimited CSV file

#### Character set

UTF-8

#### Description

These data describe the spatial and temporal characteristics from 232 trawls, as well as trawl catches represented by absolute and relative abundance of 36 fish families belonging to 13 orders of the infraclass Teleostei. The taxonomic membership of ~ 46,000 fish individuals was identified at the family level. The dataset is in Suppl. material 9.

**Data set 9.** 

Column label	Column description
Trawl	Trawl name code.
Waterbody	River and branch name.
Datetime	Trawling date and start time.
SLatitude	Trawl start latitude.
SLongitude	Trawl start longitude.
ELatitude	Trawl end latitude.
ELongitude	Trawl end longitude.
TDuration	Trawl duration, min.
Depth_mean	Mean depth, m.
Depth_min	Minimum depth, m.
Depth_max	Maximum depth, m.
Bridles	Bridles, m.
Taxa	Taxa name.
Number	Number, ind.
RNumber	Relative number, ind/h.

## Supplementary Material

7EB4B41D-3496-5FB9-9D57-2568F180CC6E 10.3897/BDJ.11.e105314.suppl1 Supplementary material 1 Water hydrological parameters Data type UTF-8 encoded file with semi-colon delimiters. Brief description Static stations physicochemical parameters. File: oo_919210.csv https://binary.pensoft.net/file/919210 Alexander I. Tsvetkov

FD2BEC55-762D-50A8-88DE-CAA3AE158797 10.3897/BDJ.11.e105314.suppl2 Supplementary material 2 Bottom trawls hydrological parameters Data type UTF-8 encoded file with semi-colon delimiters. Brief description Bottom trawls hydrological parameters. File: oo_919211.csv https://binary.pensoft.net/file/919211 Evgeniya P. Karpova and Irina U. Prusova

4FFED045-0215-5CBA-99D0-2AA842C6296F 10.3897/BDJ.11.e105314.suppl3 Supplementary material 3 Bottom fish Data type UTF-8 encoded file with semi-colon delimiters. Brief description Bottom fish families abundance and biomass. File: oo_919212.csv https://binary.pensoft.net/file/919212 Evgeniya P. Karpova and Svetlana N. Statkevich

7562905C-C651-5614-AF1E-09722FE82CEB 10.3897/BDJ.11.e105314.suppl4 Supplementary material 4 Bottom sediments Data type UTF-8 encoded file with semi-colon delimiters. Brief description Bottom sediment data. File: oo_919213.csv https://binary.pensoft.net/file/919213 Yuriy G. Udodenko and Viktor V. Zakonnov

41FD2AF3-D3D2-5033-91B1-FDC8D49E2DEF 10.3897/BDJ.11.e105314.suppl5 Supplementary material 5 Macrozoobenthos Data type UTF-8 encoded file with semi-colon delimiters. Brief description Benthic invertebrates abundance and biomass. File: oo_919214.csv https://binary.pensoft.net/file/919214 Alexander A. Prokin and Alexander I. Tsvetkov

8D0EB30A-A447-5683-9040-BDEDA097C419 10.3897/BDJ.11.e105314.suppl6 Supplementary material 6 Phytoplankton Data type UTF-8 encoded file with semi-colon delimiters. Brief description Phytoplankton biomass and total abundance. File: oo_919219.csv https://binary.pensoft.net/file/919219 Andrey N. Sharov

09FEF1A9-66BD-5C9A-84C8-4E96AB28ED17 10.3897/BDJ.11.e105314.suppl7 Supplementary material 7 Microorganisms Data type UTF-8 encoded file with semi-colon delimiters. Brief description Microorganisms quantitative data. File: oo_919364.csv https://binary.pensoft.net/file/919364 Dmitriy B. Kosolapov and Natalya G. Kosolapova

179AB581-1BB6-5147-9E01-7E9D6F75DCB2 10.3897/BDJ.11.e105314.suppl8 Supplementary material 8 Zooplankton Data type UTF-8 encoded file with semi-colon delimiters. Brief description Zooplankton biomass and total abundance. File: oo_919365.csv https://binary.pensoft.net/file/919365 Svetlana M. Zhdanova and Irina Yu. Prusova

7CD23559-F439-564C-9595-D9C62DDEA593 10.3897/BDJ.11.e105314.suppl9 Supplementary material 9 Pelagic fish assemblage Data type UTF-8 encoded file with semi-colon delimiters. Brief description Pelagic tawls characteristics and fish families abundance. File: oo_919366.csv https://binary.pensoft.net/file/919366 Mikhail I. Malin and Inga P. Malina

## Figures and Tables

**Table 1.** Summary of sampling effort to measure water hydrological parameters and biodiversity in the Mekong Delta, Vietnam. Samples were taken from 89 static sampling points unless otherwise stated (e.g. by bottom or pelagic trawling).

Assessment and taxonomic coverage	No. Observations	No. Samples	Collector(s)	Time of sampling	Filename
Water hydrological parameters	7839	926	Alexander I. Tsvetkov	December and April 2018, December 2019	Suppl. material 1
Bottom trawls hydrological parameters	4709	535 (from bottom trawling)	Evgeniya P. Karpova and Irina U. Prusova	2018-2021 (Secchi disk Feb – June 2020)	Suppl. material 2
Bottom fish	~ 88,000	535 (from bottom trawling)	Evgeniya P. Karpova and Svetlana N. Statkevich	2018-2021	Suppl. material 3
Bottom sediments	770	70	Yuriy G. Udodenko and Viktor V. Zakonnov	2019	Suppl. material 4
Macrozoobenthos	236	36	Alexander A. Prokin and Alexander I. Tsvetkov	April and December 2019	Suppl. material 5
Phytoplankton	389	35	Andrey N. Sharov	December 2018	Suppl. material 6
Microorganisms	1545	50	Dmitriy B. Kosolapov and Natalya G. Kosolapova	December 2018	Suppl. material 7
Zooplankton	1471	73	Svetlana M. Zhdanova and Irina Yu. Prusova	December 2018 and April 2019	Suppl. material 8
Pelagic fish assemblage	~ 46,000	232 (from pelagic trawling)	Mikhail I. Malin and Inga P. Malina	2019-2022	Suppl. material 9
